# Deep learning for deterioration prediction of COVID-19 patients based on time-series of three vital signs

**DOI:** 10.1038/s41598-023-37013-3

**Published:** 2023-06-20

**Authors:** Sarmad Mehrdad, Farah E. Shamout, Yao Wang, S. Farokh Atashzar

**Affiliations:** 1 Department of Electrical and Computer Engineering, New York University (NYU), New York, USA; 2 Department of Biomedical Engineering, New York University (NYU), New York, USA; 3 Division of Engineering, New York University Abu Dhabi (NYUAD), Abu Dhabi, UAE; 4 Computer Science and Engineering, New York University (NYU), New York, USA; 5 Department of Mechanical and Aerospace Engineering, New York University (NYU), New York, USA

**Keywords:** Electrical and electronic engineering, Computational science, Computer science, Signs and symptoms

## Abstract

Unrecognized deterioration of COVID-19 patients can lead to high morbidity and mortality. Most existing deterioration prediction models require a large number of clinical information, typically collected in hospital settings, such as medical images or comprehensive laboratory tests. This is infeasible for telehealth solutions and highlights a gap in deterioration prediction models based on minimal data, which can be recorded at a large scale in any clinic, nursing home, or even at the patient’s home. In this study, we develop and compare two prognostic models that predict if a patient will experience deterioration in the forthcoming 3 to 24 h. The models sequentially process routine triadic vital signs: (a) oxygen saturation, (b) heart rate, and (c) temperature. These models are also provided with basic patient information, including sex, age, vaccination status, vaccination date, and status of obesity, hypertension, or diabetes. The difference between the two models is the way that the temporal dynamics of the vital signs are processed. Model #1 utilizes a temporally-dilated version of the Long-Short Term Memory model (LSTM) for temporal processes, and Model #2 utilizes a residual temporal convolutional network (TCN) for this purpose. We train and evaluate the models using data collected from 37,006 COVID-19 patients at NYU Langone Health in New York, USA. The convolution-based model outperforms the LSTM based model, achieving a high AUROC of 0.8844–0.9336 for 3 to 24 h deterioration prediction on a held-out test set. We also conduct occlusion experiments to evaluate the importance of each input feature, which reveals the significance of continuously monitoring the variation of the vital signs. Our results show the prospect for accurate deterioration forecast using a minimum feature set that can be relatively easily obtained using wearable devices and self-reported patient information.

## Introduction

The significant shock imposed by the novel coronavirus (COVID-19) pandemic fundamentally challenged the delivery and management of health care services globally^1^. According to the World Health Organization, more than 620 million patients have been diagnosed with COVID-19 as of October 2022, and there are around 6.52 million deaths^2^. Since March 2020, 96.2 million patients have been admitted to emergency departments across the United States^3^.

Patients with COVID-19 can experience rapid deterioration entailing the need for invasive measures associated with high morbidity or mortality^4^. During the pandemic, patient prognosis was challenging, especially in the early days when the knowledge about the disease was limited, and any modifications in admission protocols could significantly alter the patient outcomes^1^. This highlighted the importance of routine patient monitoring to ensure that patients with the highest risk of deterioration receive early attention^5^.

Due to the saturation of healthcare systems and concerns over unnecessary exposure, many outpatients or those in nursing centers were advised to monitor symptoms remotely and report through telemedicine^6^. Hence, patients would avoid visiting emergency care facilities unless symptoms were considered significantly severe and would require immediate and specialized attention^7^. Although this practice could reduce exposure and unnecessary loads on emergency services^8^, it could also result in poor patient prognosis. In fact, for some patients, especially those with comorbidities, the development of symptoms was followed by sudden, drastic, and unexpected deterioration resulting in morbidity, even after discharge from a clinic^9^.

Considering the scale of data gathered from a plethora of patients with COVID-19 admitted to emergency departments worldwide, many Deep Learning (DL) and Machine Learning (ML) methods were developed for early diagnosis^10^, patient severity assessment^11^, or prognosis prediction^12,13^. These methods include the use of a Convolutional Neural Network (CNN), Variational Auto-Encoder (VAE), Gradient Boosting Machine (GBM), Multi-Layer Perceptron (MLP), Decision Tree (DT), Generalized Linear Model (GLM), Support Vector Machine (SVM), and Long-Short Term Memory (LSTM), to name a few. The datasets used in the aforementioned studies can range from Electronic Health Records (EHR) and Clinical and Comorbidity characteristics (CCC) to imaging data such as Chest X-Ray (CXR) and Computed Tomography (CT) Scan. In Table 1, we summarize relevant work based on the choice of datasets and models for the different prediction tasks. Due to the large volume of research, it would not be possible to cite all relevant papers; hence Table 1 provides a balanced list.

While most of the existing work focuses on diagnosing COVID-19 rather than patient prognosis, many studies heavily rely on large input feature sets, specifically high-dimensional imaging, such as chest CT or X-ray scans, and other non-imaging modalities, such as laboratory test results. In addition, many existing models do not exploit variations in data over time. Even though such data for computational models have shown great potential, there is a lack of seamlessly-scalable models based on minimal feature sets collected over time. We specifically prioritize data that can be collected not only in hospitals but also in nursing centers or patient homes, such as using wearable devices, e.g., smartwatches^14^.

**Table 1:** Summary of related work.

Paper	Task	Data	Machine learning model
^15–36^	Diagnosis	CXR, CT Scan, EHR, CCC	CNN, GBM, VGG19, APACHE, ResNet50, VAE, LSTM
^37–42^	Severity assessment	CXR, CCC	SVM, DenseNet, CNN, MLP
^43–47^	Prognosis	CXR, CT Scan	CNN, DenseNet121-FPN, GLM, GBM, XGBoost, DT, AlexNet, Inception-V4

Overview of related work on the diagnosis of patients with COVID-19, patient severity assessment, and patient prognosis.

In this study, we propose two deep neural networks to model time series of three vital signs only to predict the deterioration amongst patients with COVID-19. The ultimate goal of this work is to provide a light and scalable prediction model to support clinical decision-making for a wide range of patients in the long term, including at-home patients, outpatients, and inpatients. To minimize the size of the input feature space, we focus on three basic vital signs, namely, oxygen saturation (SpO2), heart rate (HR), and temperature. This design choice is motivated by the wide availability of wearable devices, such as smart watches, that can monitor these vital signs. We specifically exclude other vital signs, such as blood pressure, because they cannot be measured using readily available wearable systems.

To develop and evaluate the porposed models, we use real-world data collected at NYU Langone Health between January 2020 and September 2022. The model predicts deterioration at time horizons of 3 to 24 h using the vital-signs time-series data collected in the 24 h preceding the time of prediction (corresponding to the beginning of the prediction horizon), defined as in-hospital mortality, admission to the intensive care unit (ICU), or intubation. We refer to the sequential vital-sign data as SEQ data in the remainder of this paper. The model is also provided with a small set of features reflecting CCC, including sex, age, vaccination status, vaccination date, and status of obesity, hypertension, and diabetes (referred to as non-SEQ data).

The first model includes a temporally-dilated LSTM (TD-LSTM) network^48^ to process the SEQ data and an MLP that combines the last hidden state of the LSTM and the non-SEQ data. The LSTM network utilizes temporal dilation to enable access to more extended memory dynamics without exponentially increasing the size and complexity of the computational framework. The second model utilizes a residual temporal convolutional network (TCN)^49,50^ for SEQ data processing, and similar to the first model, an MLP is used to combine the output of the residual TCN blocks with the non-SEQ data. TCNs will preserve the causality of the time-series signal while using exponentially dilated convolutions to effectively extract features from the input signal’s full spectrum. For each prediction horizon, a separate model is trained and optimized using the focal cross-entropy^51^ loss through a three-phase training procedure.

The results show that, in the 3 to 24 h prediction time horizons the model based on TCN achieves an Area Under the Receiver Operating Curve (AUROC) of 0.8842–0.9237. While the results are not directly comparable to those in existing work due to differences in data pre-processing, the model achieves a comparable performance. For example, the model in Ref.^52^, which uses CXR images and other clinical variables, achieves 0.765 AUROC in predicting deterioration within 24 h. In order to assess the significance of the various CCC features and the importance of the temporal history of vital signs, we also perform a sensitivity analysis through an occlusion experiment. Overall, our work highlights the feasibility of achieving high model performance for deterioration prediction amongst patients with COVID-19 using minimal feature sets, which are easy to obtain not only in the hospital setting but potentially also in nursing centers and at patient homes. The following remarks are added to further clarify the main focus of this paper.

- *Remark #1* This paper report on an investigation that has shown for the first time that with deep temporal processing of only three vital signs and a set of patient’s clinical characteristics, computational models (such as those reported in this paper based on TD-LSTM and TCNs) have the power to predict the health deterioration of the patients with COVID-19 ahead of time. This paper also investigates the contribution and importance of each of the vital signs and a set of comorbidity. The outcome can have a significant impact on clinical and therapeutic management in hospitals. It should be noted that this paper does not claim to propose a new neural network architecture or a training optimization algorithm, as it is out of the scope of this paper. Rather we focus and report on the possibility of health deterioration prediction using only three vital signs processed by the proposed deep neural network architectures.
- *Remark #2* The dataset used in this study pertains to the patients with COVID-19 positive tests, but the COVID-19 infection may not necessarily be the primary reason for their hospital admission. Also, it is not possible to isolate the reason for deterioration as COVID-19 can play a compounding effect besides other comorbidities, the primary reason for admission, and underlying conditions altogether could result in deterioration. This paper does not claim to generate a general model for any abnormal health condition; instead, specifically, we focus on patients with positive COVID-19 tests.
- *Remark #3* The proposed models in this paper are trained on COVID-19-positive patients, and the paper suggests that three major vital signs can be processed by the proposed models to predict the deterioration of COVID-19-positive patients. The paper does not claim that the proposed models can be used for other respiratory illnesses, as this would require different data collection and possibly different architecture designs and training, which is out of the scope of this paper.

## Methodology

### Dataset

All methods were carried out in accordance with relevant guidelines and regulations. In this retrospective study, we use the “NYU Langone De-identified COVID-19 database^53^” collected from patients at the NYU Langone facilities between January 2020 and September 2022. The COVID-19 De-identified Clinical Database is a de-identified dataset of clinical activity at NYU Langone Health obtained from Epic starting January 1st, 2020. The data has been stripped of unique identifiers and dates have been shifted by an arbitrary number of days for each patient, which means that these data are not subject to HIPAA restrictions on research use, and thus IRB was exempted. More information can be found here^53^.

### Data pre-processing

We define inclusion and exclusion criteria. First, in the case of multiple patient encounters, we use the patient’s most recent encounter. Then, we include patients who either tested positive for COVID-19 at the facility or were already diagnosed with COVID-19 at the time of their admission. Next, we include in-patients with vital sign measurements. The vital signs of these patients, including SpO2, temperature, and HR, are periodically measured and recorded roughly every 4–5 h. For each patient, age, sex, vaccination status and time, and the presence of comorbidities, including obesity, diabetes, and hypertension, are also recorded.

Similar to previous work (see^52^ and reference therein), we define deterioration as the occurrence of the composite outcome of mortality, ICU admission, or intubation, i.e., any of the three events. For patient encounters with several adverse events, we only consider the occurrence of the earliest deterioration event. It should be noted that if there are multiple deteriorations of the same type (e.g., ICU admission) recorded for a patient more than a week apart, we only consider the latest as the reference time of deterioration for the patient. For patients who had deteriorated, we extracted vital sign data in the 48 h preceding the time of deterioration.

We use this data to define “positive” windows for each prediction horizon, where $t=0$ represents the end of the window and $t=-\,24$ represents the start of the window, such that, for example, in the prediction horizon of 24 h, deterioration would have occurred at $t=24$.

For patients who did not experience deterioration and were discharged, we use the 48 h window preceding the last vital-sign recording and similarly use those to formulate the “negative” windows. We exclude all samples containing less than 48 h of vital-sign monitoring, either preceding the deterioration time or discharge time.

**Figure 1:**
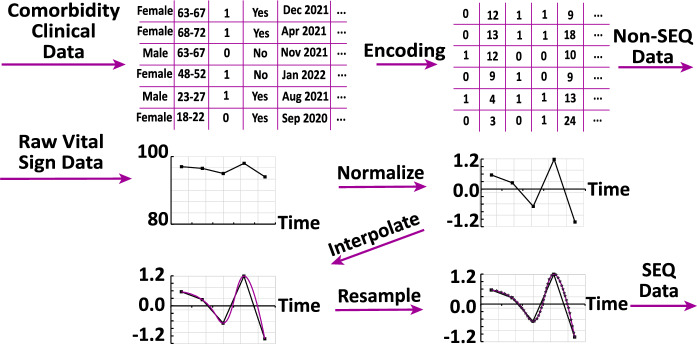
Data pre-processing pipeline. We encode the non-SEQ data and pre-process the SEQ data: (i) normalize via Z-score normalization, (ii) model the time-series using cubic spline interpolation, (iii) and resample at every 15 min.

To pre-process the time-series data, we first normalize the data using Z-score normalization based on each vital sign’s mean and standard deviation. Since the vital signs are measured at irregular intervals, we resample each time series to obtain regularly sampled input for the LSTM and TCN networks. This resampling is done by first interpolating the raw non-uniformly sampled data through cubic spline interpolation and then sampling the interpolated signal every 15 min. In Fig. 1, we show a schematic summarizing the pre-processing of the raw time-series data, which we refer to as the SEQ data.

As for the non-SEQ data, we encode patient sex, vaccination status, hypertension status, and obesity status as binary (0 or 1). For diabetes status, we use one-hot encoding to represent if a patient is non-diabetic ([1, 0, 0]), diabetic without complications ([0, 1, 0]), or diabetic with complications ([0, 0, 1]). We grouped the age into 18 different sub-groups and replaced each age with the corresponding age sub-group (value between 1 and 18). For vaccination time, we count the number of elapsed months between the time of the second COVID-19 vaccination shot and the day of the time of prediction ($t=0$).

**Figure 2:**
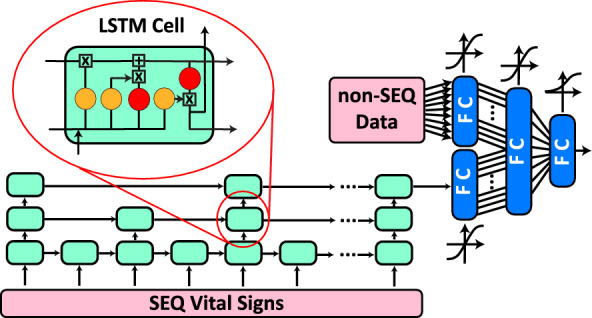
Architecture of the LSTM-based deep neural network. The SEQ vital sign network processes the SEQ data through an LSTM-based network, whereas the non-SEQ module encompasses the non-SEQ data.

**Figure 3:**
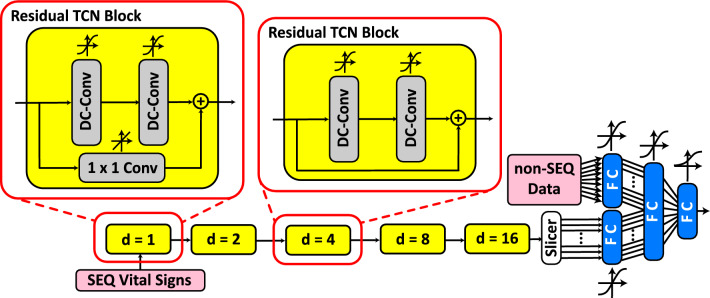
Architecture of the TCN-based deep neural network. The SEQ vital sign network processes the SEQ data through a series of residual TCN blocks followed by a FC layer, while the non-SEQ module processes the non-SEQ data.

### DL-based deterioration prediction model

We choose to develop and compare the performances of a LSTM-based model and a TCN-based model, because these two model architectures are commonly used for modeling time series data. Our proposed LSTM-based deep neural network architecture consists of TD-LSTM layers and fully-connected (FC) layers. The overall architecture of this network is shown in Fig. 2, and we refer to it as the *Recurrent Sequential Vital Sign Network (RSVS-Net)*, consisting of two modules. The SEQ data is processed by a module comprised of an LSTM network and a single FC layer, while the non-SEQ data is processed by a second module consisting of an independent FC layer. The final prediction is based on both modalities.

The TCN-based network proposed in this study includes residual TCN blocks, and FC layers. We refer to this model as *Convolutional Sequential Vital Sign Network* (*CSVS-Net*). The module that is processing the SEQ data in this network consists of a series of five residual TCN blocks with exponentially enlarging kernel dilations, and a single FC layer. The non-SEQ data processing module of the *CSVS-Net* is the same as the one in *RSVS-Net*. The architecture of *CSVS-Net* is shown in Fig. 3. For brevity, we refer to the residual TCN blocks as R-blocks throughout the rest of this paper.

#### RSVS-Net architecture details

LSTM networks are well-known for their ability to learn from SEQ data and have been widely used in studies where the time-series data is integral to the learning of the system and predicting future events^55,56^. The temporal module consists of a temporally-dilated LSTM, which takes three vital signs as input at each time step, and has three layers, each containing 32 hidden units. The final hidden state of the LSTM network, consisting of a dimensionality of 32, is processed by an FC layer with an output dimensionality of 16. The non-SEQ data, with a dimensionality of 9, is processed by a single FC layer, which computes an output of dimensionality of 16.

Finally, the latent representations of the two modalities are concatenated as a vector of dimensionality of 32 and then processed by an FC fusion layer with an output dimensionality of 8. This is then followed by a single FC layer with sigmoid activation and an output dimensionality of one, which represents the prediction that a sample precedes deterioration or is not within the specified time horizon. All of the FC layers use hyperbolic tangent activation except for the last layer, which uses sigmoid activation. The *RSVS-Net* model has 22,209 trainable parameters.

#### CSVS-Net architecture details

TCN models have gained increasing attention recently for time-series data analysis^49,50,57–59^. In TCNs, convolution is causal, meaning that the convolution output at time *t* will only rely on the signal at time *t* or earlier^49^. In the *CSVS-Net*, the SEQ data module comprises five serialized R-blocks. Each block has two dilated causal 1D convolutional (DC-Conv) layers. In addition, each R-block has a residual connection, so the network would become more effective in learning the extracted features and the modifications applied to the input signal by the DC-Conv layers. At the end of each R-block structure, the output of the last DC-Conv layer and the R-block’s input will be summed. Therefore, these signals should have matching dimensions. To ensure this criteria is satisfied throughout the model, in the first residual R-block, a $1 \times 1$ convolutional layer with linear activation function is implemented in the residual connection to match the input data dimension to the output of the DC-Conv layers.

In *CSVS-Net*, all of the DC-Conv layers in the R-blocks have 28 filters, and use hyperbolic tangent activation function. Hence, the output dimension of every R-block is ($N \times 28$), with *N* being the number of samples for each feature in the vital sign time-series signals. For 24-h observation period for each feature, since we have 4 samples in each hour, the length of the signal will be $N = 1 + (4 \times 24) = 97$. For analyzing the full length of this signal, the architecture of the *CSVS-Net* should be able to have a receptive field ($R_{field}$) big enough to analyze the entire signal length. The $R_{field}$ parameter can be computed using Eq. (1).

1 $$\begin{aligned} R_{field} = 1 + 2\times (K - 1)\times \sum _{i=1}^B d_i. \end{aligned}$$

In Eq. (1), *K* represents the filter size, *B* is the number of R-blocks in the architecture, and $d_i$ is the kernel dilation used at each R-block. By choosing $K=3$, $B=5$, and $d_i = 2^{(i-1)}$, we will have enabled the model to gain receptive field of 125. It should be noted that any other chosen number of R-blocks will either result in an insufficient or unnecessarily large receptive fields for the model. Finally, a “slicer” layer is implemented at the end of the R-block chain, which only passes through the last array of the final R-block’s output. Hence, the dimension of the slicer layer output is ($1 \times 28$). Thereafter, this $1 \times 28$ vector is processed by an FC layer with dimensionality of 16. The rest of the layers in the *CSVS-Net* are the same as the ones in *RSVS-Net*. The *CSVS-Net* model has 22,709 trainable parameters.

#### Three-phase training strategy

In order to optimize the performance of the proposed network, the training strategy consists of three phases, as described below.

- Phase 1: *Training of SEQ module* In the first phase, we train the SEQ module only and freeze all other layers. The output of the FC layer in the SEQ module is connected to another FC layer that computes the prediction. After this training phase, the weights are used to pre-initialize the SEQ module in the next phase, and we remove the second FC layer used to compute predictions during pre-training in the first phase.
- Phase 2: *Training of fusion layer* In the second phase, we compute the representations of the SEQ data after initializing the associated module with the weights obtained in the first phase, and then freeze the SEQ module. We then train the FC layer, FC fusion layer, and the FC output prediction layer using the non-SEQ data.
- Phase 3: *End-to-end fine-tuning of RSVS-Net and CSVS-Net*In the last phase, we initialize the parameters of the entire network using the weights obtained in the first two phases. The network is trained end-to-end, to improve the overall network performance.

#### Model training and evaluation

To train and evaluate the models, we divide the entire dataset into training, validation, and testing sets, with percentages of $66.6\%$, $16.6\%$, and $16.6\%$, respectively. Each dataset has the same distribution of positive and negative samples. The final performance results are obtained by evaluating the trained models on the test dataset.

We train the models using the training set for 200 epochs using the three-phase training strategy. For the first and second phases, we choose a learning rate of 0.0001 based on initial experimentation and a learning rate of 0.00001 for the final fine-tuning stage. For all phases, we use the ADAM optimizer^60^ with $\beta _1 = 0.9$, $\beta _2 = 0.999$, $\epsilon = 10^{-8}$. After training for 200 epochs, the best model is selected based on the epoch with the least validation loss across all epochs. We evaluate the models on the test set. We use the binary focal cross-entropy loss^51^ in order to manage class imbalance in the dataset. We evaluate the models’ performance using three widely used metrics for binary classification: accuracy (with a 0.5 threshold to convert the predicted probability into binary), AUROC, and Area Under the Precision-Recall Curve (AUPRC).

## Results and discussion

### Patient cohort

**Figure 4:**
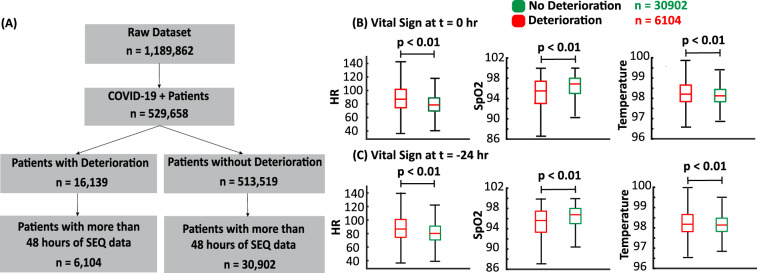
Application of data inclusion and exclusion criteria and distribution of vital signs. **(A)** In this flowchart, we illustrate the application of the inclusion and exclusion criteria, where *n* represents the number of patients after each step. **(B)** The boxplot of the vital signs recorded from the patients at the end of the 24-h input window (t = 0) corresponds to the prediction time. **(C)** The boxplot of the vital signs recorded from the patients at the beginning of the 24-h input window (t = − 24). We observe differences between the two groups (evaluated using the T-test), which motivates the design of the proposed temporal model.

**Figure 5:**
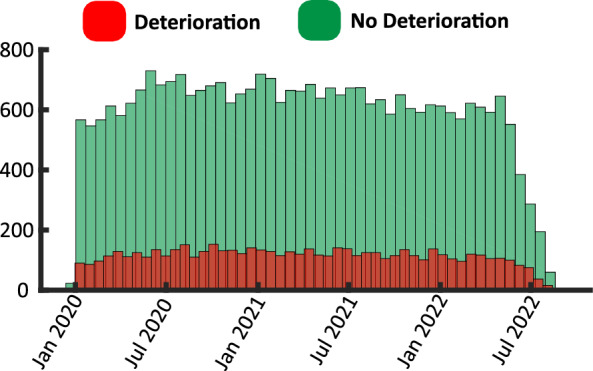
Distribution of samples over time. We show the number of patients who deteriorated vs. those who did not deteriorate in our final filtered dataset (n = 37,006).

In Fig. 4A, we show the application of the inclusion and exclusion criteria. This resulted in 37,006 patient samples, including 6104 positive samples and 30,902 negative samples. Table 2 summarizes the characteristics of the patients. In Fig. 4B, we show the differences in vital signs of the two cohorts at $t=0$, while in Fig. 4C, we show the differences in vital signs between the two cohorts at $t=-\,24$. Using t-test statistical analysis, it can be seen that the difference between the two cohorts is statistically significant even as early as 24 h before deterioration. This motivates the use of the proposed DL to decode the hidden pattern differentiating the two cohorts. In Fig. 5, we show the distribution of the admitted patients over time.

**Table 2:** Overview of patient cohort.

Characteristics	Deterioration	No deterioration
Patient, n	6104	30,902
Age (years), mean (SD)	66.0 (18.4)	63.1 (18.0)
Sex (females), n (%)	2539 (41.5)	17792 (57.5)
Diabetes w/o complications, n (%)	1510 (24.7)	5582 (18.0)
Diabetes w/ complications, n (%)	114 (1.8)	401 (1.2)
Hypertension, n (%)	2721 (44.5)	11,748 (38.0)
Vaccination n (%)	2388 (39.1)	16,776 (54.2)
Time since last vacination (months), mean (SD)	− 0.67 (6.2)	− 0.94 (7.3)
Obesity, n (%)	1003 (16.4)	5370 (17.3)
Vital signs feature sets		
SpO2 (%), mean (SD)	95.2 (4.5)	96.2 (2.7)
HR (Beats per minute), mean (SD)	93.98 (27.1)	82.9 (18.57)
Temperature ($^\circ$ F), mean (SD)	98.37 (1.6)	98.2 (1.4)

We summarize in this table the patient characteristics, including demographics, and distribution of vital signs, for patients who deteriorated and patients who did not deteriorate.

### Model performance

**Figure 6:**
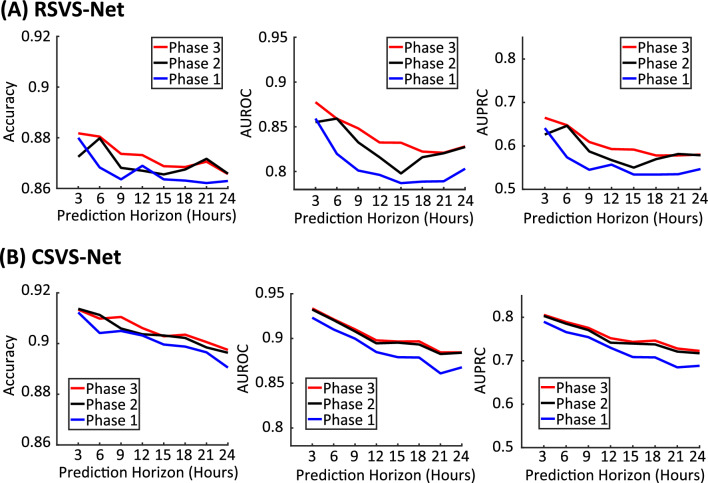
Performance results after each phase in the training strategy across all prediction horizons.

The final model performances are summarized in Table 3 and also shown in Fig. 6 after the three-phase training strategy. The performance of the *RSVS-Net* at the 24 h time horizon reaches 86.67% accuracy, 0.8278 AUROC, and 0.5805 AUPRC. Moreover, *CSVS-Net* achieves 89.75% accuracy, 0.8844 AUROC, and 0.7227 AUPRC, at the 24-h time horizon, significantly outperforming the LSTM-based model. As expected the prediction accuracy improves as the prediction horizon reduces. We suspect that the convolution-based model (*CSVS-Net*) outperformed the LSTM-based model (*RSVS-Net*) because the former was able to extract and exploit features from all the vital signs in the observation window relatively equally, while the LSTM model may reduce the contribution of the far away samples. As shown in Fig. 6, AUROC and AUPRC consistently improve at all prediction horizons after each training phase. By comparing the performance of the three phases of training, it can be observed that the adopted three-phase training strategy boosts the model’s performance by forcing the network to extract information initially from the SEQ data and, in the end, from a combination of SEQ and non-SEQ data. The improvement can be seen in accuracy, AUROC, and AUPRC. Although the datasets used in similar studies^52^ are not the same, the results are comparable to those reported in Ref.^52^ previously for a sub-number of the patients, even though the size of the input space was much larger in previous studies through the inclusion of medical imaging data.

**Table 3:** Model performance.

Prediction horizon (h)	3	6	9	12	15	18	21	24
RSVS-Net								
Accuracy	0.8826	0.806	0.8733	0.8741	0.8688	0.8689	0.8706	0.8667
AUROC	0.8754	0.8595	0.8481	0.8328	0.8322	0.8225	0.8205	0.8278
AUPRC	0.6565	0.6492	0.6098	0.5936	0.5908	0.5786	0.5783	0.5805
CSVS-Net								
Accuracy	0.9134	0.9098	0.9105	0.9061	0.9028	0.9035	0.9006	0.8975
AUROC	0.9336	0.9215	0.9102	0.8979	0.8966	0.8968	0.8844	0.8844
AUPRC	0.8056	0.7892	0.7757	0.7519	0.7434	0.7464	0.7282	0.7227

Performance summary of the proposed networks for three phases of training at all horizons.

### Ablation studies

In order to understand the impact of our design choices within the model architecture, we compare our models to two other networks. The first model, referred to as *Memory-Less Vital Sign Network (MLVS-Net)*, processes the non-SEQ data and only the last set of vital signs collected from the patient, ignoring any sequential information. Hence, instead of using a TD-LSTM or R-blocks, we process the vital-sign data (3 features) with an MLP consisting of two FC layers with an output dimensionality of 16 each. The computed representation of the MLP is then concatenated with the representation of the non-SEQ data. We train the model in a similar fashion using the three-phase training strategy, and we freeze the weights of the MLP network in phase two. The second model, referred to as the *non-Sequential Health Status Network (nSHS-Net)*, only considers the non-SEQ data. Hence, the output of the FC layer is processed by a second FC layer to generate the prediction.

**Figure 7:**
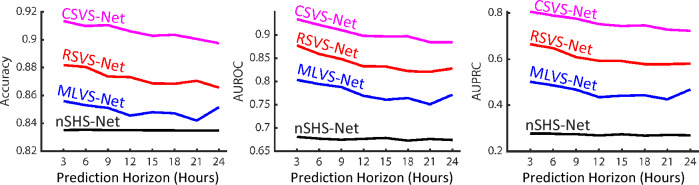
Ablation study results. Performance results for *RSVS-Net* and *CSVS-Net* (Input: non-SEQ data and SEQ vital sign data), *MLVS-Net* (Input: non-SEQ data and a single set of vital signs), and *nSHS-Net* (Input: non-SEQ only).

We compare the three models in Fig. 7. First, we observe that *nSHS-Net* performs the worst, implying that incorporating vital signs is crucial for the model prediction. When comparing *MLVS-Net* to *RSVS-Net* and *CSVS-Net*, we observe a better performance with our proposed models across all prediction horizons and evaluation metrics. This implies that incorporating sequential information can significantly improve the model’s capability in predicting deterioration relative to using a single measurement of vital signs. As already shown in Fig. 6, among the two proposed models, the *CSVS-Net* significantly outperforms the *RSVS-Net*. The numerical results of Fig. 7 are summarized in Appendix I.

**Figure 8:**
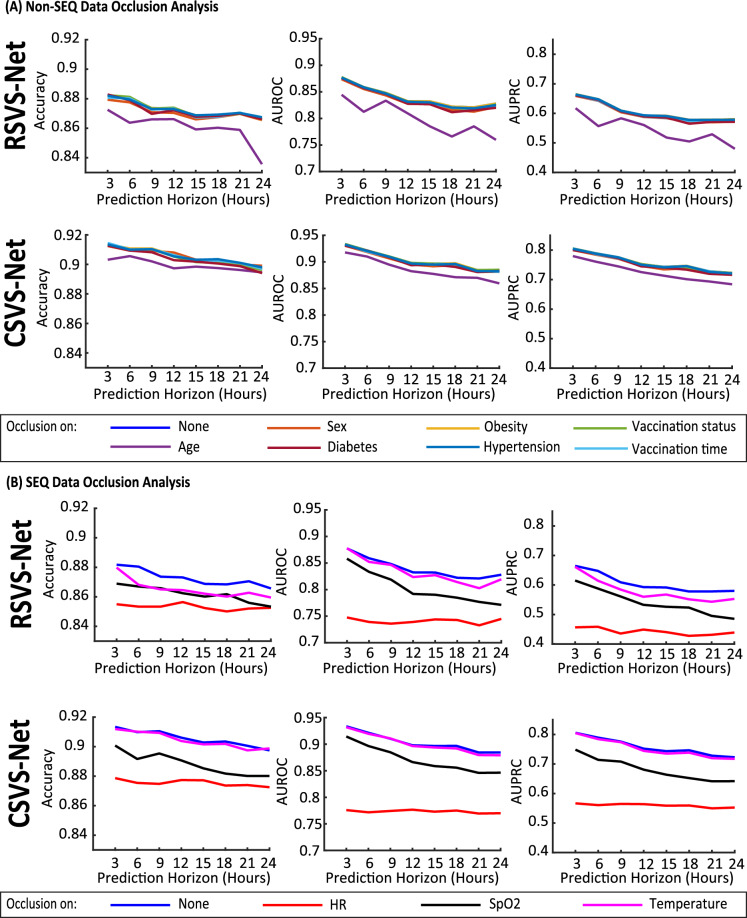
Occlusion analysis: **(A)** Occlusion of the clinical and comorbidity characteristics. Observation: Occlusion of Age decreases performance more significantly than others. **(B)** Occlusion of SEQ vital sign data. Observation: the HR contributes more significantly to the model performance than the SpO2 and temperature.

**Figure 9:**
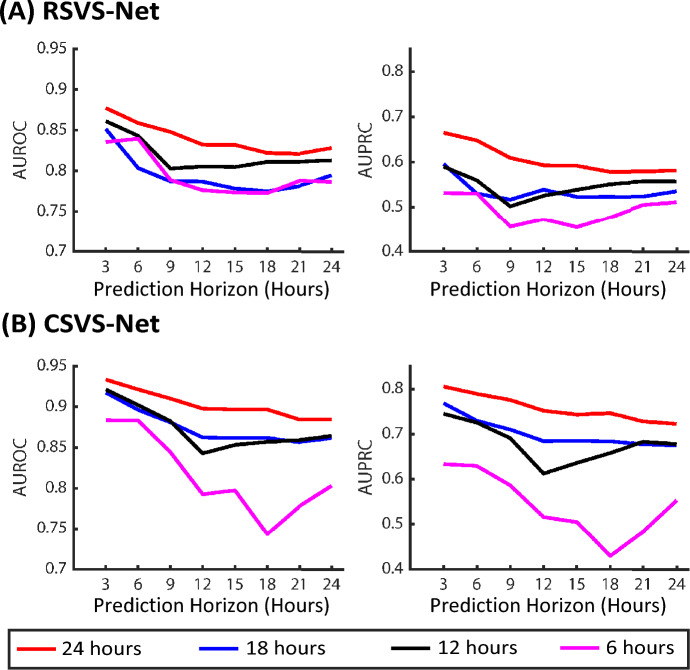
Various observation windows (6 to 24 h). The legend of the figures indicates the observation duration. Performance results of the *RSVS-Net* and *CSVS-Net* given different observation duration.

### Occlusion analysis

In order to assess the influence of each input variable, we conduct an occlusion analysis on the final optimized model. In particular, we occlude one feature at a time (by setting the corresponding values to zero) and evaluate the performance on the test set. The greater the reduction in the performance metrics upon feature occlusion, the more important the feature is for the prediction. The results of this analysis are shown in Fig. 8. As shown in Fig. 8A, amongst the non-SEQ features, we observe that age plays the most significant role in the model’s performance. The other CCC features have less impact once occluded and do not have consistent trends across the different performance metrics and prediction horizons. It should be noted that due to the correlation between the various features, some features may be relevant to the prediction task yet are not considered to be important by this occlusion analysis. For example, if one CCC feature affects the variations in the vital signs over time, then the model would capture the corresponding effect. In this case, the occlusion analysis may show that this CCC feature is unimportant. However, when a feature shows low sensitivity through the occlusion study, the need for that feature to be given to the model as an “independent” input is insignificant. Among the SEQ features, for both models, we observe heart rate as the most important feature, followed by SpO2 and temperature. For *CSVS-Net*, the temperature occlusion shows minuscule effect in the model performance. This is an interesting observation, possibly because the *CSVS-Net* model was able to capture the information encoded in the change in temperature using the observations on the change in the heart rate and/or SpO2. In other words, the inter-dependencies between the vital signs could be the cause of this observation. It should be highlighted that in Fig. 8, we have shown that the vital signs are more important than the clinical and comorbidity features, and SEQ vital signs bring additional information than the current vital signs only. The observation here matches the aforementioned analysis. Numerical results of Fig. 8 are given in Supplementary Appendix I.

### Experimenting with shorter observation windows

In the previous section, we show the importance and significance of the time history of the vital signs for predicting the deterioration of COVID-19 patients, considering an observation window of 24 h. It is also important to evaluate the behavior of the proposed model for different windows of observation. Thus, here, we analyze system performance for three shorter windows of observation (with lengths of 6 h, 12 h, and 18 h). It should be noted that for this analysis, the proposed *RSVS-Net* and *CSVS-Net* models, which were trained by observing 24 hours of patient data, were “evaluated” for 6, 12, and 18 h of the observation period. Both *RSVS-Net* and *CSVS-Net* models were not retrained for these shorter windows of observation in order to evaluate the performance of a single model for various input windows. The results of this analysis are shown in Fig. 9. As can be seen, by decreasing the duration of the observation window, the performance drops. The drop in performance caused by the shorter observation is likely caused by the lack of information used in the model to predict the deterioration event ahead. This analysis further highlights the importance of continual and prolonged monitoring, which may motivate using smart wearable medical systems.

### Comparison with COVID-19 negative patient data

**Figure 10:**
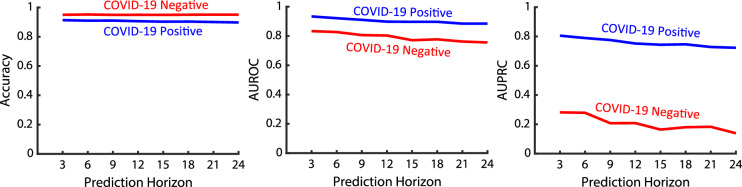
Performance comparison of *CSVS-Net* trained on COVID-19 positive and COVID-19 negative cohorts: Observation: The accuracy and AUROC metrics of the model trained on the COVID-19 negative cohort are comparable to those of the model trained on the COVID-19 positive cohort, while the AUPRC is significantly lower in comparison.

**Figure 11:**
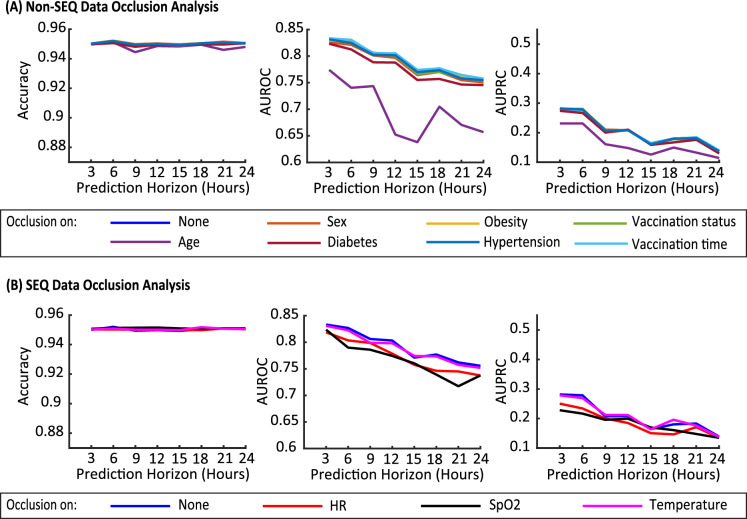
Occlusion analysis for COVID-19 negative patient cohort: **(A)** Occlusion of the clinical and comorbidity characteristics. Observation: Occlusion of Age decreases performance more significantly than others. **(B)** Occlusion of SEQ vital sign data. Observation: SpO2 vital sign has more impact on the model performance than HR and temperature.

In order to further evaluate the performance of *CSVS-Net*, we trained this model on the dataset containing the same set of SEQ and non-SEQ biomarkers derived from the patient cohort without the positive COVID-19 diagnosis (as seen in Fig. 4A). This dataset includes 2508 deteriorated and 48395 non-deteriorated patient data after pre-processing (mentioned in “Data pre-processing” section). The ratio of non-deteriorated to deteriorated patients in this dataset is noticeably higher than the COVID-19 positive dataset, which corroborates the effect of COVID-19 on patient health deterioration. The performance of the *CSVS-Net* model on this new dataset and the occlusion analysis are presented in Figs. 10 and 11, respectively. It can be seen that although the accuracy of this model is slightly higher than the *CSVS-Net* model trained on the COVID-19 positive patient dataset, AUROC follows a lower trend in comparison, and AUPRC is significantly lower. The low AUPRC conveys that the proposed model is not able to secure the same level of sensitivity to differentiate between the to-be-deteriorated and to-be-not-deteriorated subjects, as it is able to do so for the COVID-19-positive patient data. This behavior can stem from the heterogeneity of the COVID-19-negative patient data, which can point out that health deterioration prediction should be investigated using disease-specific models; also, it may highlight that a generic model may not be able to secure the needed sensitivity. In other words, the results show that even though the three proposed biomarkers (heart rate, SpO2, and temperature) can provide a sensitive prediction of deterioration for COVID-19-positive patients, they are not able to secure the same level of sensitivity for a generic patient population.

The results shown in Fig. 11A suggest that among non-sequential biomarkers, occlusion of age has the highest effect on the performance of the model trained on the COVID-19-negative patient cohort. This is the same case for COVID-19-positive patients for the proposed *CSVS-Net* model.

Moreover, in contrast to the *CSVS-Net* model trained on the COVID-19 positive dataset, the most significant SEQ vital sign that affects the model’s performance when trained on COVID-19 negative dataset is SpO2, followed by heart rate and temperature, respectively, as seen on Fig. 11B. These results, compared with the results presented in Fig. 8B, show that the occlusion of heart rate will result in a more significant drop in the performance for COVID-19-positive cases, which may suggest that the temporal dynamics of the heart rate and the corresponding temporal variations are distinctly more critical for detecting health deterioration for COVID-positive patients than for COVID-19-negative patients. Given this observation, the heart rate may be considered as a potentially unique biomarker for COVID-19 health deterioration prediction using the proposed *CSVS-Net* model.

## Conclusion

This study is motivated by the high availability of personal medical devices, such as wearable systems (e.g., smart watches), that can record time-series medical data and the prospects of using such devices in the context of telehealth to predict deterioration. In summary, we propose, develop, and evaluate a deterioration prediction model using a large dataset (n = 37,006) collected at NYU Langone Health during the period of January 2020 to September 2022. The *CSVS-Net* achieves a high AUROC of 0.8844–0.9336 in 3 to 24 hours prediction horizons and outperformed the *RSVS-Net*. Our study has several strengths. First, the models use a minimal input feature set consisting of a time series of three routine vital signs, i.e., SpO2, heart rate, and temperature. The models are also provided with basic patient information, including sex, age, vaccination status, vaccination date, and presence of obesity, hypertension, and diabetes, which can be easily collected. Compared to previous work^52^ that achieved 0.765 AUORC, our model achieves a better performance. Furthermore, our models were trained and evaluated using much smaller and more accessible data modalities (excluding any sophisticated imaging, such as CT scans). We do note that the model in Ref.^52^ was trained with significantly fewer data points (number of patients) than ours, and hence this can be another reason on the difference in performance. In addition, we also performed a sensitivity analysis through an occlusion experiment and an ablation study on various inputs including the vital signs, to assess the significance of the various clinical and comorbidity features and the importance of using time-series vital-signs data. The results showed the importance of modeling the temporal variations of vital signs and the possibility of achieving high prognosis accuracy without the need for sophisticated medical imaging. Finally, the proposed framework is scalable as it can be extended for other prediction horizon ranges and observation periods.

The limitations of the proposed work include the following. First, our observation windows for the vital signs are tested only for 6 to 24 h. We showed that reducing the window lengths leads to degraded performance. In future work, we are interested in evaluating if further increasing the observation window would significantly improve the prediction accuracy. Second, we acknowledge that the final results can be further improved via hyperparameter tuning, including the number of network layers, the number of feature channels, and convolution kernel sizes, and this is an area of future work.

To conclude, this study highlights the feasibility of an accessible and scalable model to help assist the medical workforce in decision-making. The proposed model’s versatility is important, as the data types needed for predicting the deterioration can be easily acquired from patients using wearable sensors and a few clinical data features that can be self-reported.

## Supplementary Information

Supplementary Information.

## Data Availability

The database that supports the outcomes of this paper was obtained from the NYU Langone Health’s Medical Center Information Technology (MCIT). This database is available to the NYU Langone Health community to encourage exploration of the COVID-19 patient population for exploratory research, hypothesis testing, and identification of cohorts. Restrictions do apply to the public availability of the database. Data access is upon reasonable request and requires permission from NYU Langone Health (more relevant information can be found here^53,54^). The processed data, including figures and results supporting the conclusion made in this study, are given in the paper and can be made available upon reasonable requests from the authors.

## Code availability

The deep learning model and programming code for results presented in this manuscript is available from the corresponding authors upon reasonable request.
