# Supplementary material for: Deep learning for deterioration prediction of COVID-19 patients based on time-series of three vital signs
Source: Sci Rep. 2023 Jun 20;13:9968. doi: 10.1038/s41598-023-37013-3 (PMC10282033; doi:10.1038/s41598-023-37013-3)
Supplement: Supplementary file 1 — Supplementary Information. [file 41598_2023_37013_MOESM1_ESM.pdf]

Appendix I

Here we provide numerical results with respect to Figure 7 in Table 1, and results with respect to Figure 8 in Tables 2 and 3.

Table 1. Numerical comparison of CSVS-Net, RSVS-Net, MLVS-Net, and nSHS-Net for 3-24 hours of prediction horizons

**Prediction Horizon (hours)**	**3**	**6**	**9**	**12**	**15**	**18**	**21**	**24**
**Accuracy**								
**CSVS-Net**	**0.9134**	**0.9098**	**0.9105**	**0.9061**	**0.9028**	**0.9035**	**0.9006**	**0.8975**
**RSVS-Net**	0.8826	0.8806	0.8733	0.8741	0.8688	0.8689	0.8706	0.8667
**MLVS-Net**	0.8560	0.8530	0.8511	0.8456	0.8480	0.8472	0.8420	0.8516
**nSHS-Net**	0.8352	0.8354	0.8352	0.8352	0.8350	0.8350	0.8349	0.8350
**AUROC**								
**CSVS-Net**	**0.9336**	**0.9215**	**0.9102**	**0.8979**	**0.8966**	**0.8968**	**0.8844**	**0.8844**
**RSVS-Net**	0.8754	0.8595	0.8481	0.8328	0.8322	0.8225	0.8205	0.8278
**MLVS-Net**	0.8034	0.7945	0.7879	0.7692	0.7609	0.7643	0.7511	0.7716
**nSHS-Net**	0.6811	0.6772	0.6750	0.6769	0.6787	0.6728	0.6766	0.6742
**AUPRC**								
**CSVS-Net**	**0.8056**	**0.7892**	**0.7757**	**0.7519**	**0.7434**	**0.7464**	**0.7282**	**0.7227**
**RSVS-Net**	0.6565	0.6492	0.6091	0.5936	0.5908	0.5786	0.5783	0.5805
**MLVS-Net**	0.5016	0.4864	0.4677	0.4353	0.4413	0.4434	0.4257	0.4687
**nSHS-Net**	0.2776	0.2765	0.2750	0.2690	0.2746	0.2682	0.2713	0.2700

Table 2. Numerical comparison of all the occlusions on clinical and comorbidity characteristics and SEQ vital sign data for 3-24 hours of prediction horizons for *RSVS-Net*

**Prediction Horizon (hours)**	**3**	**6**	**9**	**12**	**15**	**18**	**21**	**24**
				**Accuracy**				
**None**	0.8826	0.8806	0.8733	**0.8741**	**0.8688**	0.8689	**0.8706**	0.8667
**Sex**	0.8793	0.8775	0.8709	0.8704	0.8660	0.8675	0.8697	0.8655
**Obesity**	0.8818	0.8805	**0.8736**	0.8732	**0.8688**	0.8685	**0.8706**	0.8657
**Age**	0.8725	0.8637	0.8660	0.8662	0.8592	0.8603	0.8589	0.8357
**Diabetes**	**0.8829**	0.8790	0.8697	0.8722	0.8678	0.8683	0.8701	0.8660
**Hypertension**	0.8821	0.8795	0.8730	0.8730	**0.8688**	**0.8693**	0.8704	**0.8675**
**Vac. Time**	0.8824	**0.8813**	0.8733	0.8740	0.8683	0.8680	0.8699	0.8668
**Vac. Status**	0.8806	0.8803	0.8727	0.8733	0.8673	0.8676	**0.8706**	0.8663
**HR**	0.8550	0.8534	0.8534	0.8564	0.8524	0.8501	0.8521	0.8526
**SpO2**	0.8689	0.8670	0.8659	0.8624	0.8602	0.8618	0.8560	0.8534
**Temperature**	0.8798	0.8681	0.8650	0.8646	0.8621	0.8602	0.8628	0.8595
				**AUROC**				
**None**	0.8754	**0.8595**	0.8481	**0.8328**	**0.8322**	**0.8225**	0.8205	0.8278
**Sex**	0.8737	0.8554	0.8434	0.8275	0.8270	0.8160	0.8127	0.8214
**Obesity**	0.8774	0.8589	**0.8482**	0.8324	0.8321	0.8222	**0.8209**	**0.8280**
**Age**	0.8445	0.8125	0.8334	0.8094	0.7852	0.7660	0.7851	0.7597
**Diabetes**	0.8757	0.8572	0.8459	0.8276	0.8267	0.8117	0.8155	0.8200
**Hypertension**	0.8767	0.8586	0.8468	0.8312	0.8303	0.8204	0.8191	0.8251
**Vac. Time**	**0.8776**	0.8588	0.8477	0.8316	0.8297	0.8198	0.8174	0.8265
**Vac. Status**	0.8761	0.8572	0.8460	0.8296	0.8284	0.8202	0.8186	0.8240
**HR**	0.7475	0.7391	0.7359	0.7391	0.7439	0.7428	0.7328	0.7448
**SpO2**	0.8582	0.8334	0.8187	0.7918	0.7903	0.7847	0.7768	0.7713
**Temperature**	0.8777	0.8521	0.8465	0.8235	0.8271	0.8142	0.8026	0.8193
				**AUPRC**				
**None**	0.6565	**0.6492**	**0.6098**	**0.5936**	0.5908	**0.5786**	**0.5783**	**0.5805**
**Sex**	0.6589	0.6421	0.6036	0.5876	0.5834	0.5708	0.5696	0.5718
**Obesity**	0.6647	0.6476	0.6091	0.5928	**0.5916**	0.5781	0.5781	0.5800
**Age**	0.6173	0.5566	0.5831	0.5604	0.5179	0.5047	0.5291	0.4799
**Diabetes**	0.6612	0.6453	0.6062	0.5895	0.5855	0.5648	0.5712	0.5710
**Hypertension**	0.6637	0.6475	0.6088	0.5934	0.5906	0.5774	0.5778	0.5782
**Vac. Time**	**0.6644**	0.6467	0.6087	0.5917	0.5880	0.5749	0.5739	0.5778
**Vac. Status**	0.6614	0.6428	0.6061	0.5885	0.5841	0.5741	0.5730	0.5750
**HR**	0.4565	0.4584	0.4355	0.4490	0.4404	0.4277	0.4312	0.4389
**SpO2**	0.6150	0.5880	0.5610	0.5328	0.5261	0.5235	0.4953	0.4853
**Temperature**	0.6613	0.6145	0.5845	0.5600	0.5676	0.5515	0.5432	0.5527

Table 3. Numerical comparison of all the occlusions on clinical and comorbidity characteristics and SEQ vital sign data for 3-24 hours of prediction horizons for *CSVS-Net*

**Prediction Horizon (hours)**	**3**	**6**	**9**	**12**	**15**	**18**	**21**	**24**
				**Accuracy**				
**None**	0.9134	0.9098	0.9105	0.9061	0.9028	**0.9035**	0.9006	0.8975
**Sex**	0.9124	0.9093	0.9092	**0.9080**	**0.9032**	0.9028	0.8999	**0.8991**
**Obesity**	0.9137	**0.9108**	**0.9108**	0.9062	0.9019	**0.9035**	0.9006	0.8965
**Age**	0.9032	0.9056	0.9020	0.8973	0.8985	0.8975	0.8962	0.8946
**Diabetes**	0.9127	0.9093	0.9082	0.9028	0.9019	0.9006	0.8988	0.8941
**Hypertension**	0.9135	0.9100	0.9103	0.9054	**0.9032**	**0.9035**	**0.9011**	0.8980
**Vac. Time**	**0.9145**	0.9101	0.9103	0.9062	0.9025	0.9025	0.9009	0.8970
**Vac. Status**	0.9134	0.9095	0.9103	0.9053	0.9015	0.9011	0.8996	0.8962
**HR**	0.8787	0.8754	0.8748	0.8774	0.8772	0.8736	0.8740	0.8725
**SpO2**	0.9007	0.8916	0.8954	0.8907	0.8853	0.8818	0.8801	0.8801
**Temperature**	0.9119	0.9101	0.9093	0.9038	0.9015	0.9019	0.8975	0.8988
				**AUROC**				
**None**	0.9336	**0.9215**	0.9102	0.8979	0.8966	0.8968	0.8844	0.8844
**Sex**	0.9304	0.9186	0.9066	0.8950	0.8917	0.8938	0.8811	0.8812
**Obesity**	0.9328	0.9209	0.9097	0.8979	0.8958	**0.8976**	**0.8846**	0.8846
**Age**	0.9178	0.9101	0.8949	0.8825	0.8774	0.8712	0.8700	0.8596
**Diabetes**	0.9305	0.9212	0.9086	0.8937	0.8952	0.8905	0.8809	0.8830
**Hypertension**	0.9326	0.9206	0.9096	0.8970	0.8948	0.8961	0.8827	0.8826
**Vac. Time**	0.9320	0.9195	0.9090	0.8966	0.8951	0.8957	0.8824	0.8814
**Vac. Status**	**0.9337**	**0.9215**	0.9103	**0.8982**	**0.8968**	0.8954	**0.8846**	**0.8852**
**HR**	0.7759	0.7718	0.7743	0.7767	0.7730	0.7751	0.7695	0.7701
**SpO2**	0.9144	0.8966	0.8848	0.8663	0.8588	0.8558	0.8460	0.8464
**Temperature**	0.9319	0.9196	**0.9108**	0.8965	0.8938	0.8920	0.8795	0.8791
				**AUPRC**				
**None**	**0.8056**	**0.7892**	**0.7757**	0.7519	**0.7434**	0.7464	**0.7282**	**0.7227**
**Sex**	0.8000	0.7845	0.7704	0.7466	0.7345	0.7380	0.7213	0.7163
**Obesity**	0.8042	0.7883	0.7746	0.7531	0.7425	**0.7467**	0.7273	0.7225
**Age**	0.7797	0.7608	0.7446	0.7255	0.7133	0.7014	0.6937	0.6842
**Diabetes**	0.8001	0.7878	0.7725	0.7449	0.7406	0.7337	0.7195	0.7163
**Hypertension**	0.8043	0.7881	0.7751	0.7506	0.7416	0.7459	0.7262	0.7209
**Vac. Time**	0.8046	0.7864	0.7715	0.7504	0.7419	0.7454	0.7276	0.7190
**Vac. Status**	0.8054	0.7885	0.7748	**0.7526**	0.7424	0.7410	0.7265	0.7224
**HR**	0.5666	0.5606	0.5648	0.5640	0.5587	0.5594	0.5498	0.5524
**SpO2**	0.7484	0.7140	0.7081	0.6808	0.6637	0.6522	0.6414	0.6417
**Temperature**	0.8046	0.7841	0.7738	0.7443	0.7352	0.7382	0.7197	0.7173
